# The Genetic Architecture of Early Body Temperature and Its Correlation With *Salmonella Pullorum* Resistance in Three Chicken Breeds

**DOI:** 10.3389/fgene.2019.01287

**Published:** 2020-01-22

**Authors:** Xinghua Li, Changsheng Nie, Yuchen Liu, Yu Chen, Xueze Lv, Liang Wang, Jianwei Zhang, Weifang Yang, Kaiyang Li, Chuanwei Zheng, Yaxiong Jia, Zhonghua Ning, Lujiang Qu

**Affiliations:** ^1^ State Key Laboratory of Animal Nutrition, Department of Animal Genetics and Breeding, National Engineering Laboratory for Animal Breeding, College of Animal Science and Technology, China Agricultural University, Beijing, China; ^2^ Beijing Municipal General Station of Animal Science, Beijing, China; ^3^ Breeding Department, Beinongda Technology Co., LTD, Beijing, China; ^4^ Institute of Animal Sciences, Chinese Academy of Agricultural Sciences, Beijing, China

**Keywords:** *Salmonella Pullorum*, body temperature, disease resistance, heritability, genome-wide association study, chicken

## Abstract

New-born chicks are vulnerable to bacterial infections and not good at regulating body temperature. There is a close relationship between thermal regulation and immunity, however, the underlying mechanism is not well understood. *Salmonella Pullorum* (SP) is a major concern in developing countries and causes significant economic losses in poultry industry. Early body temperature (EBT) has previously shown to be correlated with host immunity and resistance to pullorum disease. In this study, we challenged three independent chick populations (Beijing You, Dwarf and Rhode Island Red) with SP at 4 days of age, and rectal temperature was measured before and after the SP attack from 2 to 7 days of age. Host defense to SP was evaluated by survival and spleen SP carrier status. The results showed that chicks with higher EBT before SP infection tend to have higher resistance to later SP attack in two populations (Dwarf and Beijing You). The association between EBT before SP attack and SP resistance was non-significant in Rohde Island Red population (*P* = 0.06), but the trend was consistent with the other two populations. We also found low to moderate heritability in all three populations for EBT before and after the SP attack ranging from 0.14 to 0.20. Genome-wide association studies identified several genomic regions and biological pathways determining EBT before SP attack, which provides candidate functional genes of this trait. Our results reveal the genetic determination of EBT, and the relationship between EBT and SP resistance, providing an alternative strategy for improving SP resistant activities in chicken.

## Introduction

Pullorum disease, an acute and systematic chick infectious disease caused by *Salmonella Pullorum* (SP), occurs frequently and is a major economic concern for chicken farms in developing countries (Barrow et al., 2012). SP can cause high mortality rates in chicks under 20 days old and yet few symptoms in adult chickens. In the poultry industry, disease resistance is an important trait with substantial economic value and disease control is drawing more and more public concerns (Cheng et al., 2013). Existing disease management is not enough to prevent disease outbreaks and genetic selection for resistant birds has been regarded as a promising complementary strategy. Difficulty in measuring disease resistance phenotypes remains a major obstacle in genetic research and breeding. This difficulty arises since it is demanding and costly to do large-scale challenge tests in farm settings. Additionally, different infection models have been used in the study of *Salmonella* resistance, complicating the interpretation of results (Calenge et al., 2010). Despite these problems, it is important to identify traits associated with SP resistance to aid in breeding and selection strategies.

Body temperature is an important physiological trait and indicator of health status (Sund-Levander et al., 2002). Young animals are unable to regulate their own body temperature and generally susceptible to various pathogen infections. There is a pronounced rise in the body temperature of chicks during the first week after hatching, particularly during the first four days (Lamoreux and Hutt, 1939). There are also breed differences of thermoregulation in newly hatched chicks (Dunnington and Siegel, 1984), indicating a genetic determination of early body temperature (EBT). Interestingly, chicken resistance to SP has been shown to be positively correlated with rapid rise of body temperature in the first 10 days post-hatch (Roberts and Cards, 1935). A two-generation selection experiment further confirmed the association between EBT and SP resistance (Hutt and Crawford, 1960a), suggesting the possibility of breeding resistant chicks based on EBT instead of pathogen exposure. In fact, many studies have reported the close relationship between body temperature and immunity regulation (Hori et al., 1991; Watanabe et al., 2008; Fisher et al., 2010; Morrison and Nakamura, 2019). However, the genetic basis of EBT has not been systematically investigated and its correlation with resistance to SP needs more detailed demonstration.

In the present study, we orally challenged three independent chicken breeds/lines with SP at 4th day post-hatch, including a highly selected commercial line (Rhode Island Red, RIR), a Chinese local line (Beijing You, BY), and a synthetic layer line (dwarf, DW) to achieve disease phenotypes. We recorded the body temperature of chicks before and after the SP challenge to better understanding the relationship between EBT and SP infection. We conducted variance component analyses to estimate genetic parameters. We also carried out genome-wide association studies to identify genomic regions and candidate genes correlated EBT before SP infection.

## Materials and Methods

### Ethics Statement

All experiments were approved by the Animal Care and Use Committee of China Agricultural University (Approval ID: XXCB-20090209). All the animals were fed and handled according to the regulations and guidelines established by this committee, and all efforts were made to minimize suffering.

### Chickens and Bacterial Challenge

The details about the chicken populations and challenge test procedure can be found in (Li et al., 2018). Briefly, 621 DW, 586 RIR, and 482 BY chicks were orally inoculated with 4.8×10^7^ CFU of SP culture at 4 days post-hatch. 40 chicks from each line were randomly selected as control group, which was mock-challenged with the same volume (0.5 mL) of phosphate buffer saline. After 36 days, all chicks still alive were killed by neck dislocation, and their spleens were removed aseptically and immediately plated onto MacConkey agar media with an inoculation loop in order to check for the presence of SP. Chicks were classed as either susceptible (dead), carriers (with detectible SP load in the spleen) or clear (without detectible SP load in the spleen).

### Body Temperature Determination

Chick temperature was estimated with clinical electronic thermometers. Thermometer probes were dipped in glycerin to facilitate insertion into the cloaca, and held in the chick rectum at 1.5 cm for 15 s before recording temperature. All temperature measurements were conducted between 13:00-16:00 from 2 to 7 days post-hatch. Early body temperature (EBT) was defined as the average temperature of 2 to 4 days post-hatch, because chick body temperature experiences a dramatic rise during the first week after hatching (Lamoreux and Hutt, 1939; Hutt and Crawford, 1960a). Temperature measurement at 4 days post-hatch was taken before SP inoculation. Fever was determined as the average temperature of 5 to 7 days.

### Heritability Analyses

Chicks in each population were designated as either susceptible, carrier or clear, which represents different degrees of SP resistance. The differential analysis among different resistance groups was carried out using PROC GLM of SAS (version 9.2; SAS Institute Inc., Cary, NC).

For our genetic analysis, SP resistance was defined as a three-state categorical trait (dead, carrier, clear), and temperature as a continuous trait. Variance and covariance components were estimated using the DMU software package (Madsen and Jensen, 2008). Bivariate analyses of resistance-EBT were performed using a bivariate threshold model, which was fitted using the RJMC module in the DMU software package. Heritabilities of EBT were estimated with AI-REML in a linear model of the DMU software package. Both models included the fixed effect of the cage and the random effects of additive genetics, as shown in matrix notation:

y=Xβ+Za+e

where y is a vector of phenotypic value; β and a are the vectors of fixed effects and random additive effects, respectively; X and Z are appropriate incidence matrices of fixed effects and random additive effects, respectively; and e is the random residuals.

### DNA Isolation, Genotyping and Quality Control

We isolated individual genomic DNA from blood or muscle samples by the classical phenol-chloroform procedure. The DNA integrity was verified by agarose gel electrophoresis and the purity was checked by A260/280 ratio using the NanoDrop 2000 spectrophotometer (Thermo Fisher Scientific™). Not all the chicks were used for genotyping. Only the individuals with conclusive symptom and high DNA quality were genotyped. Some individuals in the clearance group were also randomly abandoned to match the corresponding phenotype proportion. A total of 842 samples were sent for genotyping. A total of 842 qualified individual genomic DNA samples were genotyped using the Affymetrix 600 K chicken high-density array (Affymetrix, Inc. Santa Clara, CA, USA).

The software Axiom Analysis Suite 3.1 was used for single-nucleotide polymorphism (SNP) calling and initial quality control with the Best Practices Workflow setting from the raw data in the form of CEL files. Only samples with dish quality control (DQC) of 0.82 or better and call rate >95% were used for the downstream analyses. The SNP QC metrics were set to default values recommended by Affymetrix, except that only “PolyHighResolution” SNPs were used in our analysis. Furthermore, we excluded SNPs with unknown or repeated physical positions with an R script. SNPs on sex chromosomes were also discarded because the current statistical methods are not powerful enough to detect association between phenotypes and sex-related genotypes. After these QC steps, 818 samples and 452,291 SNPs remained. To increase the power of association analysis, we dropped 85,140 SNPs due to low variation among subpopulations (monomorphic in any one of the three breeds), 62,450 variants deviating from Hardy-Weinberg equilibrium (HWE) test (P < 1 × 10^-5^) and 1,774 SNPs with minor allele frequency (MAF) < 0.05 using PLINK v1.90 (Purcell et al., 2007). Missing genotypes were imputed based on information of the remaining SNP genotypes from each subpopulation separately, according to the software Beagle Version 4 (Browning and Browning, 2009). A total of 818 samples and 302,927 SNPs were included in the subsequent genome-wide association analysis. The detailed sample composition used in the GWAS is presented in the **Supplementary Table 1**.

### Genome-Wide Association Analyses

Population structure and relatedness are major sources of confounding effects in genetic association studies (Astle and Balding, 2009). The most popular method for GWAS containing related individuals is the linear mixed model (LMM) method due to its effectiveness in controlling population stratification bias and reducing the inflation from polygenic background (Yu et al., 2006; Kang et al., 2008; Kang et al., 2010; Zhang et al., 2010; Lippert et al., 2011; Zhou and Stephens, 2012; Loh et al., 2015). In this study, we assessed the population structure through a principal component analysis (PCA) implemented in PLINK package. Considering that SNP clusters in high linkage disequilibrium may bias the PCA results, we first pruned the full SNP set to 23,870 independent SNPs using the -indep-pairwise 25 5 0.2 command parameters in PLINK. These unlinked SNPs were then used to calculate the top three PCs used as covariates in the mixed model. Furthermore, a pairwise kinship matrix was built using the pruned SNPs.

A single-marker univariate linear mixed model was used for testing associations between early body temperature and effective SNPs. The disease phenotype was analyzed using the following model:

y=Wα+xβ+u+ϵ

In this formula, y denotes the trait values of body temperature; **W** refers to a matrix of covariates (fixed effects that contain the top three principal components, genotyping batch and a column of 1s) controlling for population structure and batch effect; α is a vector of corresponding coefficients that includes the intercept; x is a vector of the marker genotypes; **β** is the corresponding marker’s effect; **u** is a vector of random polygenic effects with a covariance structure as **u**~N (0, **K**Vg), where **K** represents a genetic relatedness matrix derived from independent SNPs and Vg is the polygenic additive variance; and ϵ is a vector of random errors. The association analysis was performed using the GEMMA v0.96 software (Zhou and Stephens, 2012). The Wald test statistic $AGB=B_{\mathrm{leaf}}+B_{\mathrm{stem}}+B_{\mathrm{panicle}}$was used to test the null hypothesis *β* = 0 for each SNP. We used the “qqman” package in R to draw the Manhattan and quantile-quantile (Q-Q) plots. Moreover, the genomic inflation factor λ was estimated using “GenABEL” package to evaluate the control for population stratification. (Aulchenko et al., 2007).

The P-value threshold of genome-wide significance was calculated through the simpleM method implemented in a R script for multiple testing correction (Gao et al., 2010). Through the simpleM calculation, a total of 72,648 independent effective tests were obtained. The genomewide and suggestive significance values were then calculated as 6.88e-7 (0.05/72,648) and 1.38e-5 (1.00/72,648), respectively.

### Candidate Genes and Functional Annotation

The gene position information was annotated using BioMart in the Ensembl database (Ensembl Genes 96). Candidate genes were retrieved within 1 Mb of the significant SNPs. To provide insight into the functional enrichment of candidate genes, we carried out GO (Gene Ontology) and Pathway analysis using Metascape (Zhou et al., 2019). Metascape is an integrated portal that combines functional enrichment, interactome analysis, gene annotation, and membership search to leverage over 40 independent databases. As the knowledge base for human is the most comprehensive, we converted the chicken genes to their human orthologs for enrichment analysis.

## Results

### Descriptive Statistics of Phenotypes

Mortality rates after SP inoculation were 25.1% for RIR, 8.3% for BY and 22.7% for DW. For RIR, 17.9% of chicks were classed as carriers, compared with 0.6% for BY and 15.8% for DW. Finally, 57% of RIR completed cleared the infection, compared with 91.1% for BY and 61.5% for DW.

**Figure 1:**
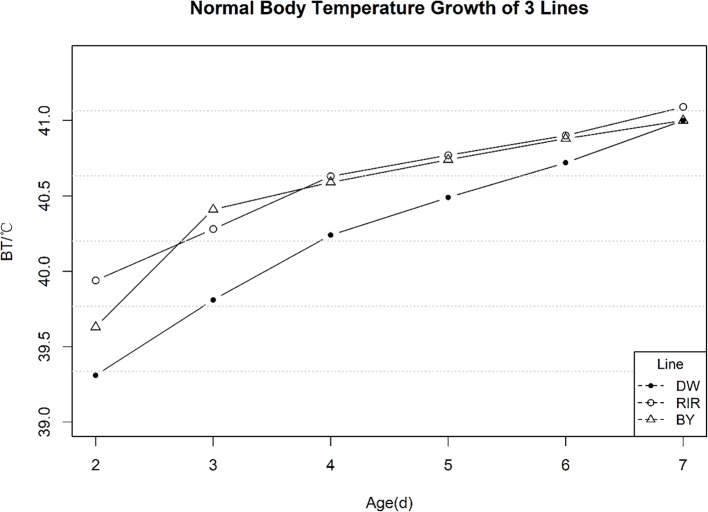
Normal Early Temperature of 3 Lines.

Normal temperature variation of the control group from 2 to 7 days post-hatch is shown in **Figure 1**. EBT increased over time in all three lines, and is particularly sharp during the first two days of measurement, consistent with earlier studies (Hutt and Crawford, 1960b).At 7 post-hatch, the average temperatures of three populations were not significantly different. The mean, standard deviation (SD), coefficient of variation (CV), minimum and maximum values of average early temperature are shown in **Table 1**. We found that the average EBT for DW chicks was 0.3–0.4°C less than the other two lines(p-value < 0.01).

**Table 1:** Early body temperature in three study lines.

Line	Mean*	SD	CV	Min	Max
RIR	40.37^a^	0.31	0.78	39.20	41.07
DW	39.92^b^	0.38	0.95	38.90	41.00
BY	40.24^a^	0.31	0.77	39.13	41.03

*The same letter at the upper right represents no significant difference (p-values > 0.05), and different letters represents significant difference (p-values < 0.01) between the means.

### Correlations Between EBT and SP Resistance

**Figure 2:**
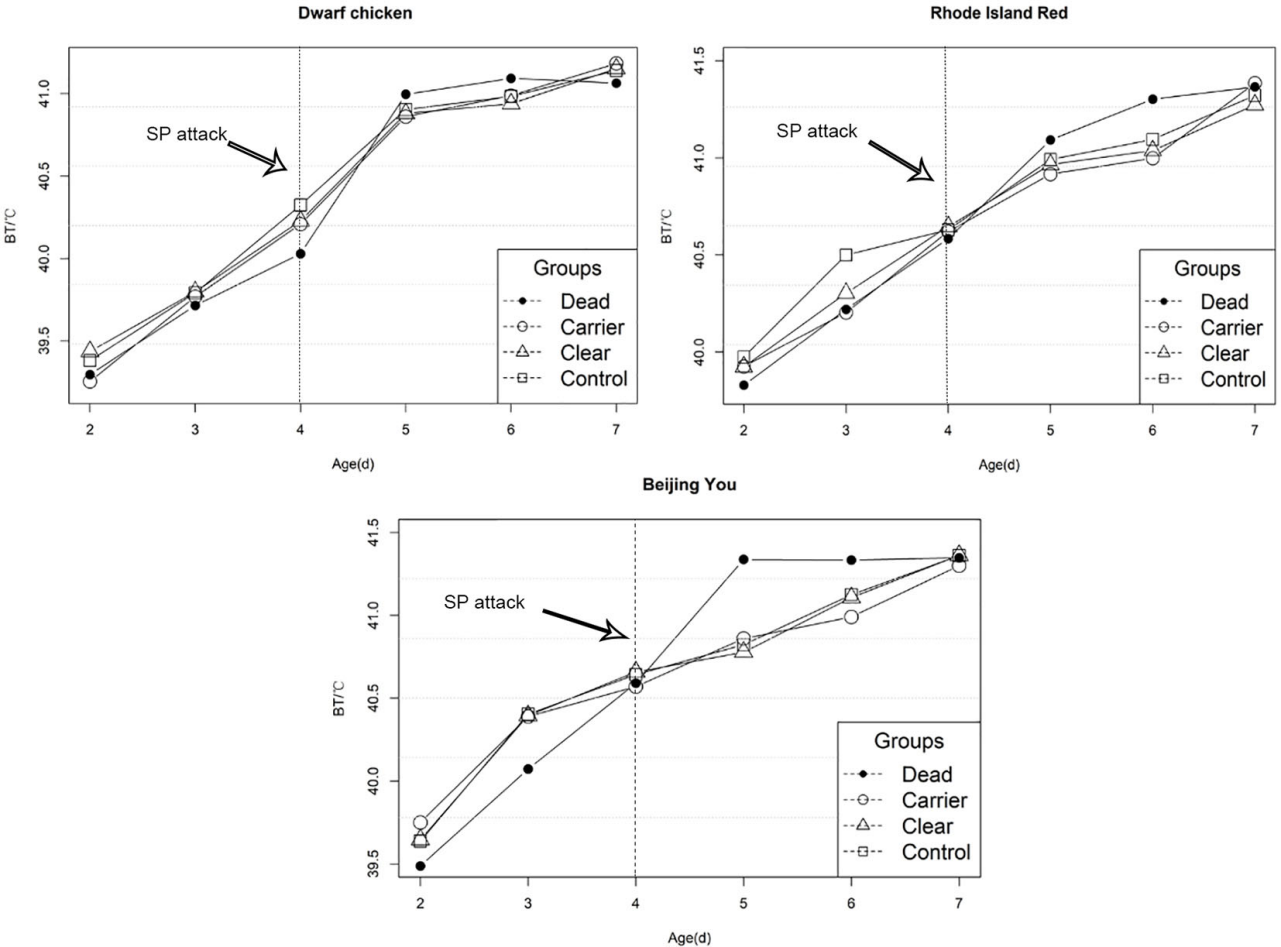
Early body temperature variation before and after salmonella infection. Chicks were challenged with SP at 4 days of age.

Differences in EBT for chicks with different SP outcomes is shown in **Figure 2**. After infection, EBT for challenged chicks increased significantly compared to the control group (p-value < 0.01).

**Figure 3:**
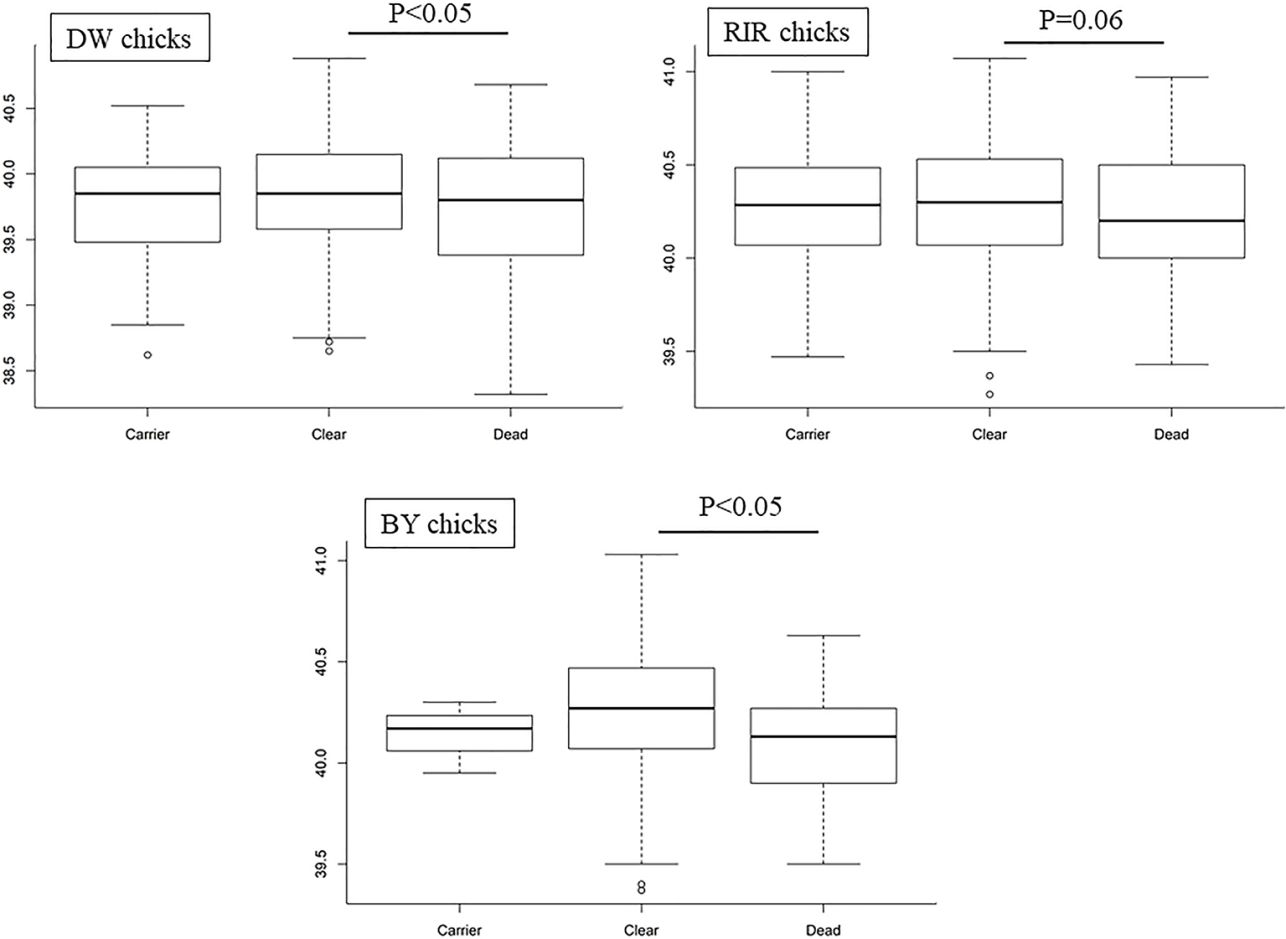
Boxplots of temperature from different *Salmonella Pullorum* resistance; P represents the p-value between Dead and Clear groups.

As shown in **Figure 3**, we found that average EBT for SP-susceptible birds was lower in all three chicken lines compared to carrier and resistant birds (p < 0.05 in DW and BY). EBT was also lower for RIR; however, the results were not statistically significant (p-value = 0.06). There was no significant difference in EBT between carrier and clear birds (p-value > 0.05).

### Heritability and Genetic Correlations

Genetic parameters are important references for the selection of infection-resistant animals in breeding. Higher heritability means that the trait could be improved through direct phenotype selection. Most disease trait showed heritabilities lower than 0.1, which means the selection could not work very well. However, we can use other traits to carry an indirect selection. The estimated heritability of EBT is shown in **Table 2**. EBT before and after SP infection shows low to moderate heritability (0.14–0.20). The genetic correlation between resistance and EBT is 0.23 in DW, 0.09 in RIR and 0.04 in BY, however the reduced correlation in BY is likely due in large part to the low overall mortality in this population. Our results suggest that selection for increased early EBT in some populations would be a useful tool to increase SP resistance.

**Table 2:** Heritability of early body temperature and fever.

Lines	Traits	Heritability	SE
DW	EBT	0.178	0.074
	Fever	0.200	0.086
RIR	EBT	0.144	0.033
	Fever	0.159	0.054
BY	EBT	0.137	0.071
	Fever	0.136	0.081

### Genome-Wide Association Studies

**Figure 4:**
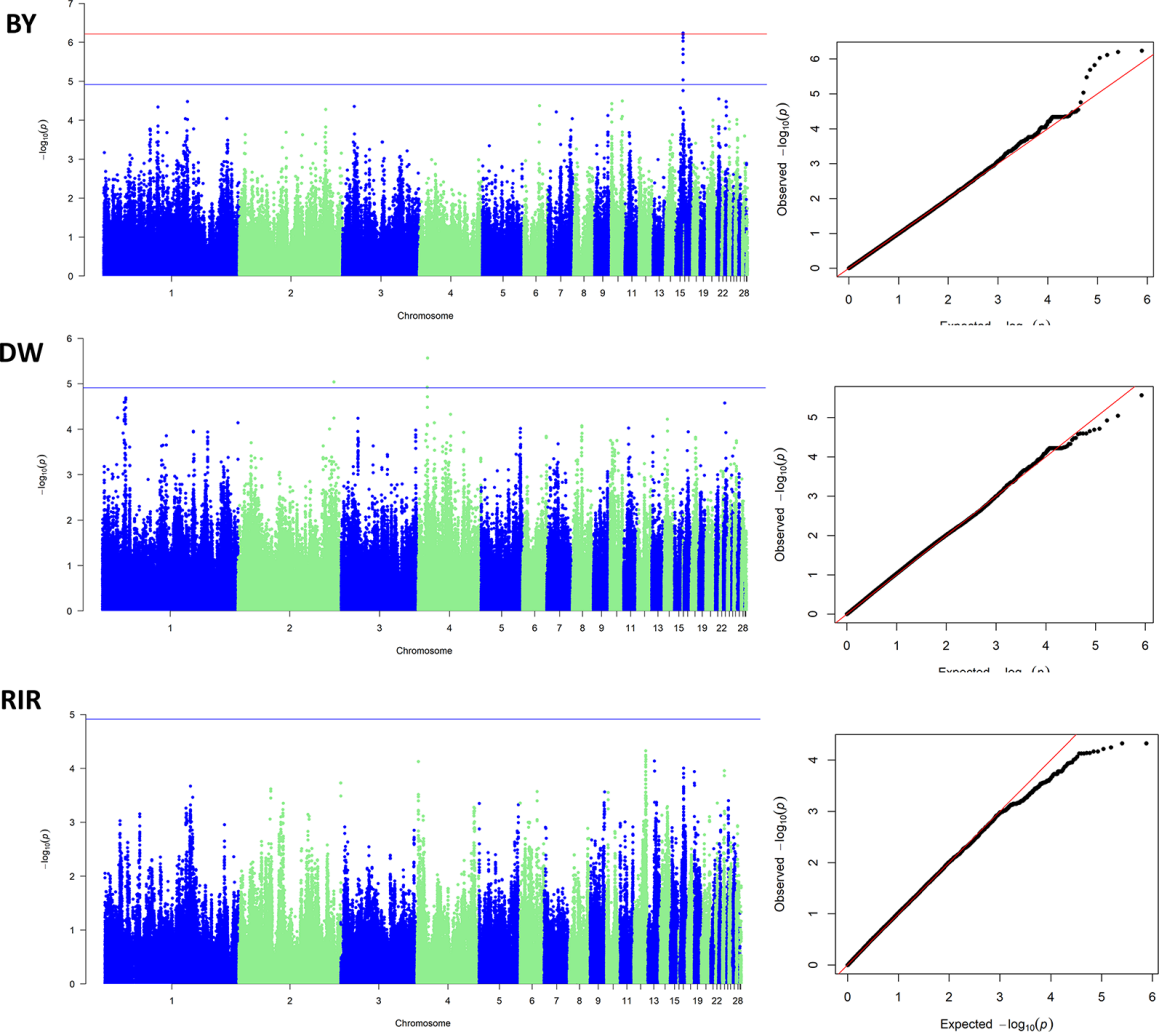
Manhattan plots and Q-Q plots of GWAS results.

Since all of the GWASs were carried out within a pure breed population, the population stratification was not a major confounding effect in our study. The inflation factors (λ) were estimated to be 0.9996, 1.0267 and 1.0093 in BY, DW and RIR respectively. The Manhattan plot and the Q-Q plot for the GWAS results are displayed in **Figure 4**. In the BY population, the single-marker association analysis identified 2 SNPs above the genome-wide significant threshold on chromosome 15. In the DW population, 3 SNPs crossing the suggestive genomewide significant threshold were identified on chromosomes 2 and 4. However, no significant SNP was identified in the RIR population. Details of the significant markers identified are presented in **Table 3**. One of the five SNPs are upstream SNP, and the other four are intron SNP.

**Table 3:** List of significant SNPs of genome-wide association studies.

Breed	SNP	Chr	Position	p-Value	Annotation
BY	Affx-50682328	15	10790585	5.74E-07	intron
BY	Affx-50682367	15	10802838	6.31E-07	intron
DW	Affx-51376528	4	13836195	2.72E-06	intron
DW	Affx-50860361	2	138881714	9.10E-06	intron

### Functional Annotation of Significant Regions

**Figure 5:**
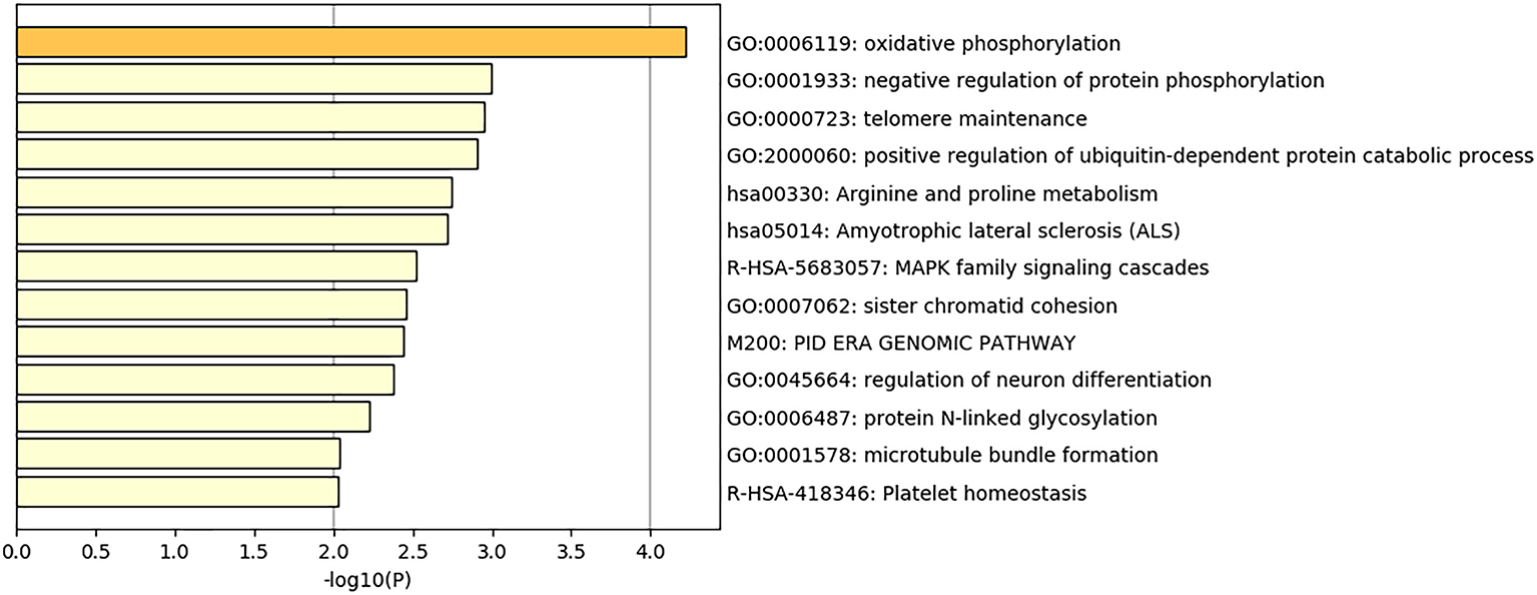
Heatmap of top 13 clusters with their representative enriched terms.

There were three significant regions detected to be associated with EBT in total. With the help of BioMart in the Ensembl database, we achieved 162 genes located within 1 Mb of the significant SNPs. These gene IDs were converted into their human orthologs, and we finally obtained 118 gene IDs which were sent to Metascape for enrichment analysis. GO and Pathway analysis were performed to determined the biological functions of these genes. 57 GO biological processes were detected, such as oxidative phosphorylation, ubiquitin-dependent protein catabolic process and MAPK family signaling cascades. Seven KEGG pathway were found, including Alzheimer’s disease, Arginine and proline metabolism, Amyotrophic lateral sclerosis (ALS) Oxidative phosphorylation, Parkinson’s disease, Non-alcoholic fatty liver disease (NAFLD), and Cardiac muscle contraction. Five Reactome gene sets were identified, including MAPK family signaling cascades, Neddylation, S Phase, MAPK6/MAPK4 signaling and Platelet homeostasis. Heatmap of top 13 clusters with their representative enriched terms is presented in **Figure 5**.

## Discussion

Thermal regulation is a basic physiological process in response to infection, which has been conserved in warm-blooded and cold-blooded vertebrates’ evolution for more than 600 million years (Evans et al., 2015). A fever response is usually beneficial for the host survival during infection, and young animals show different body temperature regulations and disease resistance. It is easy to associate these two traits which may share some genetic determination.

There has been a significant interest in the genetic basis of *Salmonella* resistance (Berthelot et al., 1998; Beaumont et al., 1999; Girard-Santosuosso et al., 2002). In the current study, we found major differences in genetic correlations between EBT and SP resistance among different chicken populations, implying that genetic background has an important influence in these traits. In both DW and BY chickens, we observe that higher EBT tends to be associated with greater SP resistance, consistent with previous research(Hutt and Crawford, 1960b). Differences in EBT could reflect the transition rate from poikilothermy to homiothermy in the chick, and may be related to better control of thermoregulation. Alternatively, the relationship between EBT and SP resistance may be related to immune defense mechanisms such as phagocytosis, bacteriolysis or the production of antibodies (Scholes, 1940).

We also provide a detailed description of host temperature dynamics following salmonella infection in chickens. The DW chicks with the sex-linked dwarf allele (*dw*) showed a lower EBT than the normal chicks, which is consistent with Dunnington’s result (Dunnington and Siegel, 1984). We found evidence of increased temperature in all populations after inoculation. After challenge, susceptible birds showed significantly greater EBT than carrier or resistant birds, suggesting that they suffered more severe infection. This is somewhat unexpected, as fever is typically associated with bacterial infection and has been shown to increase host survival (Kane and Peterson, 1976; Kluger et al., 1975). However, an acute body weight loss occurred as anticipated. Besides, we observe that both clear and carrier chicken showed longer sustained elevated EBT after infection, in contrast to susceptible chicks which showed rapid increases in EBT initially, with a decrease two days post-infection.

For the first time, we estimated the heritability of early body temperature. As showed in **Table 2**, EBT is a trait with low to moderate heritability, indicating genetic determination plays an important role. GWAS remains a promising method for studying genomic architecture of complex traits. Our study identified 4 significant SNPs associated with early body temperature in total. However, the significant regions do not overlap among different chicken breeds, showing that the genetic composition of EBT is relatively complex just as many disease traits. The most significant SNP, Affx-50682328 is located at the intron of *OSBP2* gene on GGA15. The annotation of *OSBP2* (oxysterol binding protein 2) showed it has the function of cholesterol binding and lipid transporter activity. Other positional candidate genes include several Class I MHC mediated antigen processing and presentation genes such as *FBXO21, FBXO32,* and *FBXW8*; immune-related genes such as *CARD8, DTX1,* and *TRIB1*; RNA processing genes such as *SF3A1, ZMAT5, DDX54*. In mice study, *FBXO21* was found to facilitate Lys29-linkage and activation of *ASK1* (apoptosis signal-regulating kinase 1) and promote type I interferon production upon viral infection (Yu et al., 2016). Genetic variants in *CARD8* gene have been reported to be associated with many infectious diseases such as inflammatory bowel disease (McGovern et al., 2006), Crohn’s disease (Roberts et al., 2010) and tuberculosis (Pontillo et al., 2013). *TRIB1* was reported to play a critical role in differentiation of tissue-resident M2-like macrophages (Satoh et al., 2013). These immune-related genes are very likely to connected early body temperature and SP resistance. In another chicken body temperature GWA study, Van Goor identified QTL for body temperature on GGA 14, 15, 26, and 27 (Van Goor et al., 2015). Though there are no overlapped genomic regions, our study shared a candidate gene (*DDX54*) with theirs (*DDX42*), both of which are putative RNA helicases and implicated in a number of cellular processes involving alteration of RNA secondary structure such as translation initiation, nuclear and mitochondrial splicing, and ribosome and spliceosome assembly (Linder, 2006). Interestingly, RNA splicing was recently found to be correlated with body temperature (Preußner et al., 2017), pointing out that candidate genes with similar functions deserve further research.

The present study focused on investigating the genetic determination of EBT, the correlation with SP resistance and providing positional candidate genes. More researches are necessary to explore the biological processes and molecular mechanism of these genes to associate immune activities and body temperature regulation.

## Conclusion

In conclusion, our study describes early body temperature variation before and after SP infection. Controlling for genetic background, we show that elevated EBT is associated with greater SP resistance in BY population, and that EBT and SP resistance are correlated in the BY and DW population. Our results could prove useful in designing breeding programs for improvement of SP resistance in some chicken breeds, and provide positional candidate genes for genetic determination of early body temperature.

## Data Availability Statement

The datasets generated for this study can be found in FigShare https://figshare.com/articles/EBT-GWAS_zip/10059092.

## Ethics Statement

The animal study was reviewed and approved by Animal Care and Use Committee of China Agricultural University.

## Author Contributions

LQ, ZN and YJ conceived and designed the research. XLi performed the experiments, analyzed, and interpreted the data, and wrote the manuscript. CN, YJ and YL performed the experiments and interpreted the data. YC, XLv, LW, JZ, WY, CZ and KL interpreted the data and contributed reagents and materials.

## Funding

This work was supported by the earmarked fund for the Beijing Innovation Team of the Modern Agro-industry Technology Research System (BAIC04–2018, BAIC04–2019), National Natural Science Foundation of China (31772581) and Chinese Agricultural Research System (CARS-41).

## Conflict of Interest

Author CZ was employed by company Beinongda Technology Co., Ltd, China.

The remaining authors declare that the research was conducted in the absence of any commercial or financial relationships that could be construed as a potential conflict of interest.

## Abbreviations

BY, Beijing You; DW, Dwarf Chicken; EBT, Early body temperature; GWAS, genome-wide association study; LD, linkage disequilibrium; LMM, linear mixed model; PCA, principle component analysis; RIR, Rhode Island Red; SNP, single-nucleotide polymorphism; SP, *Salmonella pullorum*.
