# Supplementary material for: The Genetic Architecture of Early Body Temperature and Its Correlation With Salmonella Pullorum Resistance in Three Chicken Breeds
Source: Front Genet. 2020 Jan 22;10:1287. doi: 10.3389/fgene.2019.01287 (PMC6987447; doi:10.3389/fgene.2019.01287)
Supplement: Supplementary file 1 [file Table_1.docx]

**Supplementary Table 1**

**Sample composition of the three chicken lines used in the GWAS**

| **Breed** | **Died** | **Carrier state** | **Clearance** | **Total** |
| --- | --- | --- | --- | --- |
| BY | 27 | 0 | 50 | 77 |
| DW | 89 | 77 | 198 | 364 |
| RIR | 84 | 84 | 209 | 377 |
| Total | 200 | 161 | 457 | 818 |
